# *Plasmodium falciparum* histidine-rich protein (PfHRP2 and 3) diversity in Western and Coastal Kenya

**DOI:** 10.1038/s41598-018-38175-1

**Published:** 2019-02-08

**Authors:** David Nderu, Francis Kimani, Kelvin Thiong’o, Evaline Karanja, Maureen Akinyi, Edwin Too, William Chege, Eva Nambati, Christian G. Meyer, Thirumalaisamy P. Velavan

**Affiliations:** 1 Institute of Tropical Medicine, University of Tübingen, Tübingen, Germany; 2 School of Health Sciences, Kirinyaga University, Kerugoya, Kenya; 3 Center for Biotechnology Research and Development, Kenya Medical Research Institute, Nairobi, Kenya; 4 Department of Biochemistry and Biotechnology, School of Biological and Life Sciences, Technical University of Kenya, Nairobi, Kenya; 5 Vietnamese-German Centre for Medical Research (VG-CARE), Hanoi, Vietnam; 6 Faculty of Medicine, Duy Tan University, Da Nang, Vietnam; 7 Fondation Congolaise pour la Recherche Médicale, Brazzaville, Congo

## Abstract

*Plasmodium falciparum* histidine-rich proteins 2 (PfHRP2) based RDTs are advocated in falciparum malaria-endemic regions, particularly when quality microscopy is not available. However, diversity and any deletion in the *pfhrp2* and *pfhrp3* genes can affect the performance of PfHRP2-based RDTs. A total of 400 samples collected from uncomplicated malaria cases from Kenya were investigated for the amino acid repeat profiles in exon 2 of *pfhrp2* and *pfhrp3* genes. In addition, PfHRP2 levels were measured in 96 individuals with uncomplicated malaria. We observed a unique distribution pattern of amino acid repeats both in the PfHRP2 and PfHRP3. 228 PfHRP2 and 124 PfHRP3 different amino acid sequences were identified. Of this, 214 (94%) PfHRP2 and 81 (65%) PfHRP3 amino acid sequences occurred only once. Thirty-nine new PfHRP2 and 20 new PfHRP3 amino acid repeat types were identified. PfHRP2 levels were not correlated with parasitemia or the number of PfHRP2 repeat types. This study shows the variability of PfHRP2, PfHRP3 and PfHRP2 concentration among uncomplicated malaria cases. These findings will be useful to understand the performance of PfHRP2-based RDTs in Kenya.

## Introduction

Malaria associated morbidity and mortality has steadily declined in recent years due to increased use of bed nets and other pertinent control measures^1,2^. Although malaria is an easily treatable parasitic disease, the rapid development of antimalarial drug resistance considerably threatens control efforts.

Microscopic examination of stained blood smears continues to serve as the gold standard for malaria diagnosis^3^. However, it is not readily available in resource-limited areas due to the scarcity of skilled personnel, reliable electricity supply, good quality reagents and infrastructure^3^. The WHO and national malaria control programmes (NMCPs) have put in place strategies to circumvent this pitfall. One of these strategies is compulsory malaria testing by appropriate test systems, including rapid diagnostic tests (RDTs) prior to the prescription of antimalarial drugs.

Discrepancies in the performance of antigen detecting tests are attributed to a combination of factors such as parasite levels, interpretation of RDT results and/or the handling and storage of RDT kits. However, some of the inconsistencies observed with results from *Plasmodium falciparum* histidine-rich protein 2 (PfHRP2)-based RDTs may also be explained by the deletion of the *pfhrp2* gene and its structural homologue, *pfhrp3*, in some parasite isolates.

In the 1990s, the first hand−held immunochromatographic malaria diagnostic test known as rapid diagnostic test (RDT), was developed to address the shortcomings of microscopy^4^. RDTs detect *Plasmodium* antigens using monoclonal antibodies (MAbs) impregnated on a nitrocellulose membrane^5^. About 10 µl of blood is required to perform the test. The antigens targeted by commercially available RDTs include *P. falciparum* histidine-rich protein 2 (PfHRP2), lactate dehydrogenase (LDH) and aldolase. PfHRP2 is *P. falciparum*-specific, aldolase is genus-specific, and LDH is available in three formats, namely *P. falciparum-*specific*, P. vivax-*specific and genus-specific^6^.

Currently, there are more than 200 commercially available malaria RDT brands. The RDTs differ between manufacturers, depending on the antigen or combination of antigens that can be detected^7^. The WHO recommendation on the RDT format to be used in a given geographical area depends on the predominant *Plasmodium* species. For regions where *P. falciparum* is predominant such as sub-Saharan Africa, the WHO recommends the use of PfHRP2-based RDTs. Eighty-three percent of RDTs procured in 2016, globally, were supplied to African countries^2^. Ninety percent of these RDTs target PfHRP2^8^.

The national malaria control programme of Kenya adopted the use of PfHRP2 detecting RDTs in 2012^9,10^. Future use of this test is threatened in many malaria-endemic areas including Kenya by the deletion of the gene coding for *P. falciparum* PfHRP2 and extensive antigen diversity that contributes to variation of the sensitivity of these tests^11–17^. There has been a considerable increase in the number of countries with *P. falciparum* isolates devoid of *pfhrp2* and/or *pfhrp3* over the last eight years. Recent entrants include Mozambique, Eritrea, Rwanda and Kenya^8,12,15,18–23^. It is therefore important to monitor parasite factors that can undermine malaria RDT-based diagnosis and, in the long run, safeguard the efficacy of antimalarial drugs and promote prompt and appropriate management of febrile illnesses.

The present study aimed to investigate the diversity of PfHRP2 and its homologue PfHRP3 as well as variation of PfHRP2 levels in uncomplicated malaria cases from two malaria-endemic regions located in Western and Coastal Kenya.

## Results

Exon 2 of both *pfhrp2* and *pfhrp3* was detected in all the 400 samples analysed in this study. Of this, 244 *pfhrp2* and 267 *pfhrp3* PCR products were successfully sequenced and their amino acid sequences deduced for an assessment of PfHRP2 and PFHRP3 diversity, respectively. The remaining 156 *pfhrp2* and 133 *pfhrp3* PCR products were excluded from further analysis because the nucleotide sequences for these amplicons could not be obtained despite repeated attempts. PfHRP2 and PfHRP3 amino acid sequence diversity among Kenyan *P. falciparum* isolates was characterised by differences in the frequency, occurrence and structural organisation of different amino acid repeat types.

### PfHRP2 diversity

A total of 228 different PfHRP2 amino acid sequences were identified among 244 PfHRP2 sequences deduced in this study. The size of PfHRP2 was between 206 and 317 amino acids. Overall, PfHRP2 had a total of 20 to 37 amino acid repeat types per isolate. The organization of the amino acid repeat types in PfHRP2 was highly diverse. Thus, 94% (214/228) of PfHRP2 sequences occurred once only. The remainder (14) were shared among 30 isolates of which 12 sequences were identified in 2 isolates and 2/14 sequences occurred in 3 isolates.

Thirteen previously reported PfHRP2 amino acid repeat types were identified in *P. falciparum* isolates from Kenya as shown in Table 1^16^. The frequency of these repeat types was similar among Western and Coastal Kenyan isolates. Table 2 shows the occurrence of PfHRP2 repeat types in this study. Repeat types 2 and 7 were identified in all isolates, whereas repeat types 1, 3, 5, 6, 8, 10 and 12 were observed in 80% to 99% of the isolates. Repeat type 4 (27%) occurred in a few isolates only. Repeat types 13 (8.2%), 14 (6.6%) and 19 (1.2%) were rare. All isolates lacked repeat types 9 and 11. Repeat type 14 did not occur in Tiwi, Coastal Kenya, and repeat type 19 was identified in only three isolates (3%) from Busia, Western Kenya. Most of the PfHRP2 repeat types had a similar occurrence within and between Western and Coastal Kenya, except for four repeat types (Table 2). Type 6 was significantly more prevalent and type 10 was significantly less prevalent in Western than in Coastal Kenya. Type 14 was significantly more prevalent in Msambweni than in Tiwi. Thirty-nine new PfHRP2 repeat types, which have not been reported previously, were identified at low frequencies with repeat type AHHAAH (5.7%) being the most common one (Table 3).

**Table 1:** Comparison of the range of individual PfHRP2 and PfHRP3 repeat types in malaria-endemic sites of Kenya.

Repeat types	Repeat sequence	PfHRP2	PfHRP3	Malaria-endemic region		Overall
				Western	Coastal	
Type 1	AHHAHHVAD	+	+	0–6	0–9	0–9
				**0–4**	**1–3**	**0–4**
Type 2	AHHAHHAAD	+	+	7–18	6–16	6–18
				**0–1**	**0**	**0**
Type 3	AHHAHHAAY	+	−	0–3	0–3	0–3
Type 4	AHH	+	+	0–6	0–6	0–6
				**1**	**1**	**1**
Type 5	AHHAHHASD	+	−	0–3	0–3	0–3
Type 6	AHHATD	+	−	0–8	0–6	0–8
Type 7	AHHAAD	+	+	2–12	1–12	1–12
				**0–1**	**1**	**0–1**
Type 8	AHHAAY	+	−	0–3	0–3	0–3
Type 10	AHHAAAHHATD	+	−	0–3	0–3	0–3
Type 12	AHHAAAHHEAATH	+	−	0–1	0–1	0–1
Type 13	AHHASD	+	−	0–2	0–2	0–2
Type 14	AHHAHHATD	+	−	0–2	0–2	0–2
Type 15	AHHAHHAAN	−	+	**0–1**	**0–1**	**0–1**
Type 16	AHHAAN	−	+	**6–18**	**9–15**	**6–18**
Type 17	AHHDG	−	+	**3–10**	**3–8**	**3–10**
Type 18	AHHDD	−	+	**1–4**	**1–3**	**1–4**
Type 19	AHHAA	+	−	0–1	0	0–1
Type 20	SHHDD	−	+	**1**	**1**	**1**

The plus (+) and minus (−) signs show the presence or absence, respectively, of individual amino acid repeats in PfHRP2 and PfHRP3. The range of individual amino acid repeats in PfHRP3 is shown in bold.

**Table 2:** Comparison of the occurrence of individual PfHRP2 repeat types within and between two malaria-endemic regions in Kenya.

Repeat types	Western				Coastal			Regional Total			Overall
	Nyando n (%)	Busia n (%)	Mbita n (%)	*p*-*value*^a^	Msambweni n (%)	Tiwi n (%)	*p*-*value*^a^	Western n (%)	Coastal n (%)	*p*-*value*^b^	Total n (%)
Type 1	30 (96.8)	116 (99.1)	24 (96.0)	ns	37 (94.9)	32 (100)	ns	170 (98.3)	69 (97.2)	ns	239 (98.0)
Type 2	31 (100)	117 (100)	25 (100)	ns	39 (100)	32 (100)	ns	173 (100)	71 (100)	ns	244 (100)
Type 3	29 (93.5)	109 (93.2)	21 (84)	ns	33 (84.6)	29 (90.6)	ns	159 (91.9)	62 (87.3)	ns	221 (90.6)
Type 4	8 (25.8)	28 (23.9)	7 (28.0)	ns	10 (25.6)	13 (40.6)	ns	43 (24.9)	23 (32.4)	ns	66 (27.0)
Type 5	25 (80.6)	91(77.8)	19 (76.0)	ns	35 (89.7)	26 (81.3)	ns	135 (78)	61 (85.9)	ns	196 (80.3)
Type 6	31 (100)	116 (99.1)	25 (100)	ns	32 (82.1)	32 (100)	**0.014**	172 (99.4)	64 (90.1)	**0.001**	236 (96.7)
Type 7	31 (100)	117 (100)	25 (100)	ns	39 (100)	32 (100)	ns	173 (100)	71 (100)	ns	244 (100)
Type 8	30 (96.8)	111 (94.9)	24 (96.0)	ns	38 (97.4)	31 (96.9)	ns	165 (95.4)	69 (97.2)	ns	234 (95.9)
Type 10	27 (87.1)	97 (82.9)	23 (92.0)	ns	38 (97.4)	31 (96.9)	ns	147 (85)	69 (97.2)	**0.007**	216 (88.5)
Type 12	30 (96.8)	100 (85.5)	24 (96.0)	ns	36 (92.3)	29 (90.6)	ns	154 (89)	65 (91.5)	ns	219 (89.8)
Type 13	2 (6.5)	9 (7.7)	3 (12.0)	ns	3 (7.7)	3 (9.4)	ns	14 (8.1)	6 (8.5)	ns	20 (8.2)
Type 14	1 (3.2)	13 (11.1)	2 (8.0)	ns	8 (20.5)	0	**0.007**	16 (9.2)	8 (11.3)	ns	24 (9.8)
Type 19	0	3 (2.6)	0	ns	0	0	ns	3 (1.7)	0	ns	3 (1.2)

The 244 isolates analysed were distributed as follows; Nyando 31, Busia 117, Mbita 25, Msambweni 39 and Tiwi 32 isolates. The superscript letters show the *p*-*values* of comparison of the occurrence of repeat types within (^a^) and between (^b^) malaria-endemic regions in Kenya. Statistically significant difference (*p* < *0.05*) shown in bold. ns: not significant.

**Table 3:** List of new PfHRP2 and PfHRP3 amino acid repeat types identified in Kenya.

PfHRP2				PfHRP3			
Repeat Types	Known repeats	Novel Repeats	(%)	Repeat Type	Known repeats	Novel Repeats	(%)
Type 1	AHHAHHVAD	AHHAHHVA**Y**	0.8	Type 1	AHHAHHVAD	AHHAHH**GAE**	0.3
		AHHAHHV**P**D	0.4			AHH**S**HHVAD	0.7
		AHH**T**HHVAD	0.4			AH**Q**AHHVAD	0.3
Type 2	AHHAHHAAD	AHHA**D**HAAD	0.4			A**Q**HAHHVAD	0.3
		AHHAHHAAH*	4.1	Type 7	AHHAAD	AHHA**D**D	0.3
		AHHAHHA**D**D	1.2	Type 15	AHHAHHAAN	AHHAHHA**P**H	0.3
		AHHAHHA**DH**	0.4	Type 16	AHHAAN	AHHAAH*	0.7
		AHHAHHA**P**D	0.8			AHHA**D**N	0.3
		AHHAHHA**PH**	0.4			AHHA**PH**	0.3
Type 4	AHH	AH**Q**	0.4			AHHA**S**N*	1.5
		A**D**H	0.4			AHH**T**AN	0.3
Type 5	AHHAHHASD	AHHA**P**HASD	0.4			AH**Q**A**D**N*	0.3
		AHH**D**HHASD	0.4			A**Y**HA**SH**	0.3
Type 6	AHHATD	AHHAT**H**	0.4	Type 17	AHHDG	AHHD**E**	0.3
Type 7	AHHAAD	AHHA**P**D	0.4			AHHD**H**	0.3
		AHHAAH*	5.7			AH**Y**DG	0.3
		AHHA**D**D*	2.0			**P**HHDG	0.7
		AHHA**H**D	0.4			**P**H**Q**DG	0.3
		AHHA**N**D	1.2			**S**HHDG	9.7
		AHHA**NH**	0.4	Type 18	AHHDD	A**P**HDD	0.3
Type 8	AHHAAY	AHHA**D**Y	0.4				
Type 10	AHHAAAHHATD	AHHAAAH**DAND**	0.4				
		AHHAAAHHA**N**D	0.8				
		AHHAAAHHAT**G**	0.4				
		AHHAA**T**HHATD	1.2				
		AHHAA**T**HHATD	0.4				
Type 12	AHHAAAHHEAATH	A**D**HAAAH**DD**AATH	0.4				
		A**D**HAAAHHEAATH	0.4				
		AHHAAAH**DDH**ATH	0.4				
		AHHAAAH**D**EAATH	0.8				
		AHHAAAHHEAA**A**H	0.8				
		AHHAAAHHEAA**S**H	0.4				
		AHHAAAHHE**S**ATH	0.4				
		AHHAAAHH**H**AATH	0.4				
		AHHAAAH**P**EAATH	0.4				
		AHHAA**P**HHEAATH	0.4				
		AHHA**D**AHH**D**AATH	0.4				
Type 13	AHHASD	AHHAS**H**	0.4				

The asterisks (*) shows new repeat types with >1 copy per isolate. The single-letter amino acid code in bold shows the position where the novel repeat types differ from the known repeat types. % shows the occurrence of the novel repeat types.

We classified *P. falciparum* isolates into groups A, B, I and C based on the product of the number of repeat type 2 and type 7 (type 2 × type 7), as described in the methods section (PfHRP2 and PfHRP3 diversity), to determine their distribution on basis PfHRP2 diversity. Our study revealed that most of the isolates were in group B (type 2 × type 7; ranges from 50 to 100) (Table 4). The occurrence of group A (type 2 × type 7; >100) was significantly higher in Mbita than in Busia and Nyando, Western Kenya. In the Coastal region, however, the occurrence of group C (type 2 × type 7; <43) was significantly higher in Tiwi than in Msambweni.

**Table 4:** Comparison of the occurrence of PfHRP2 groups (Baker model) within and between malaria-endemic regions of Kenya.

PfHRP2 groups	Western				Coastal			Regional total		*p*-*value*^b^	Overall
	Nyando n (%)	Busia n (%)	Mbita n (%)	*p*-*value*^a^	Msambweni n (%)	Tiwi n (%)	*p*-*value*^a^	Western n (%)	Coastal n (%)		Total n (%)
Group A	0	10 (8.5)	5 (20.0)	**0.025**	2 (5.1)	1 (3.1)	ns	15 (8.7)	3 (4.2)	ns	18 (7.4)
Group B	27 (87.1)	90 (76.9)	17 (68.0)	ns	30 (76.9)	21 (65.8)	ns	134 (77.5)	51 (71.8)	ns	185 (75.8)
Group I	0	8 (6.8)	2 (8.0)	ns	5 (12.8)	2 (6.3)	ns	10 (5.7)	7 (9.9)	ns	17 (7.0)
Group C	4 (12.9)	9 (7.7)	1 (4.0)	ns	2 (5.1)	8 (25.8)	**0.035**	14 (8.1)	10 (14.1)	ns	24 (9.8)

Groups A, B, I and C constitute PfHRP2 sequence whose Baker repeat type 2 × type 7 number is >100, ranges from 50 to 100, ranges from 44 to 49 and <43, respectively. The superscript letters show the *p*-*values* of the comparison group occurrence within (^a^) and between (^b^) malaria-endemic regions of Kenya. Statistically significant difference (*p* < *0.05*) shown in bold. ns: not significant.

**Figure 1:**
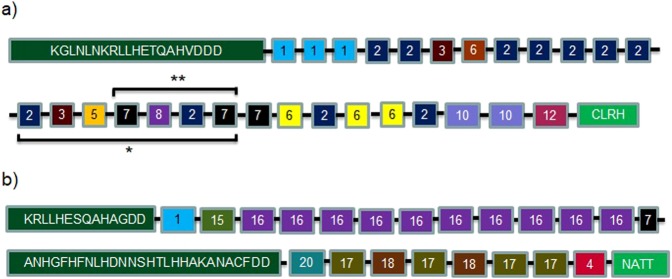
Schematic diagram of the structural organisation of PfHRPs 2 and 3 amino acid repeat types in Kenya. (**a**) *P. falciparum* histidine-rich protein 2. (**b**) *P. falciparum* histidine-rich protein 3. *Semi-conserved amino acid repeat motif; **partial amino acid repeat motif.

The structural organisation of the PfHRP2 repeat types was highly variable. Nevertheless, three characteristic features were observed. The repetitive region of most of the PfHRP2 sequences began with repeat type 1 in 96% to 99% of the *P. falciparum* isolates, ended with type 12 in 85.5% to 96.8% of *P. falciparum* isolates and had a semi−conserved PfHRP2 repeat type motif composed of repeat types 2, 3, 5, 7 and 8 in 50% (121/244) of the isolates as illustrated in Fig. 1a. Twenty−eight percent of the isolates shared parts of this motif composed of types 7, 8 and 2. These two motifs were not identified in 55 isolates.

### PfHRP3 diversity

A total of 124 different PfHRP3 amino acid sequences were deduced from 267 *pfhrp3* (exon 2) nucleotide sequences obtained in this study. The size of PfHRP3 ranged from 160 to 247 amino acids, whereas the total number of amino acid repeat types per isolate ranged between 18 and 33 types. Repeat types 1, 4 and 7 identified in PfHRP2 were also present in PfHRP3. The number of repeat types 16, 17 and 18 per isolates varied most (Table 1). Apart from type 2, which occurred only in one isolate from Nyando, the other repeat types had an occurrence of ≥97%. We identified 20 new PfHRP3 repeat types that have not been reported previously at low frequencies (Table 3). The amino acid sequence SHHDG was the most common (9.7%) novel PfHRP3 repeat type.

Structurally, PfHRP3 was more conserved than PfHRP2. Eighty-one out of 124 (65.3%) different PfHRP3 sequences occurred only once, whereas 43/124 (34.7%) different PfHRP3 sequences were shared by 2–17 isolates each. In addition, the organisation of repeats was conserved between isolates as shown in Fig. 1b. A non-repetitive sequence was located between two PfHRP3 repetitive motifs.

### PfHRP2 concentration in whole blood samples

**Figure 2:**
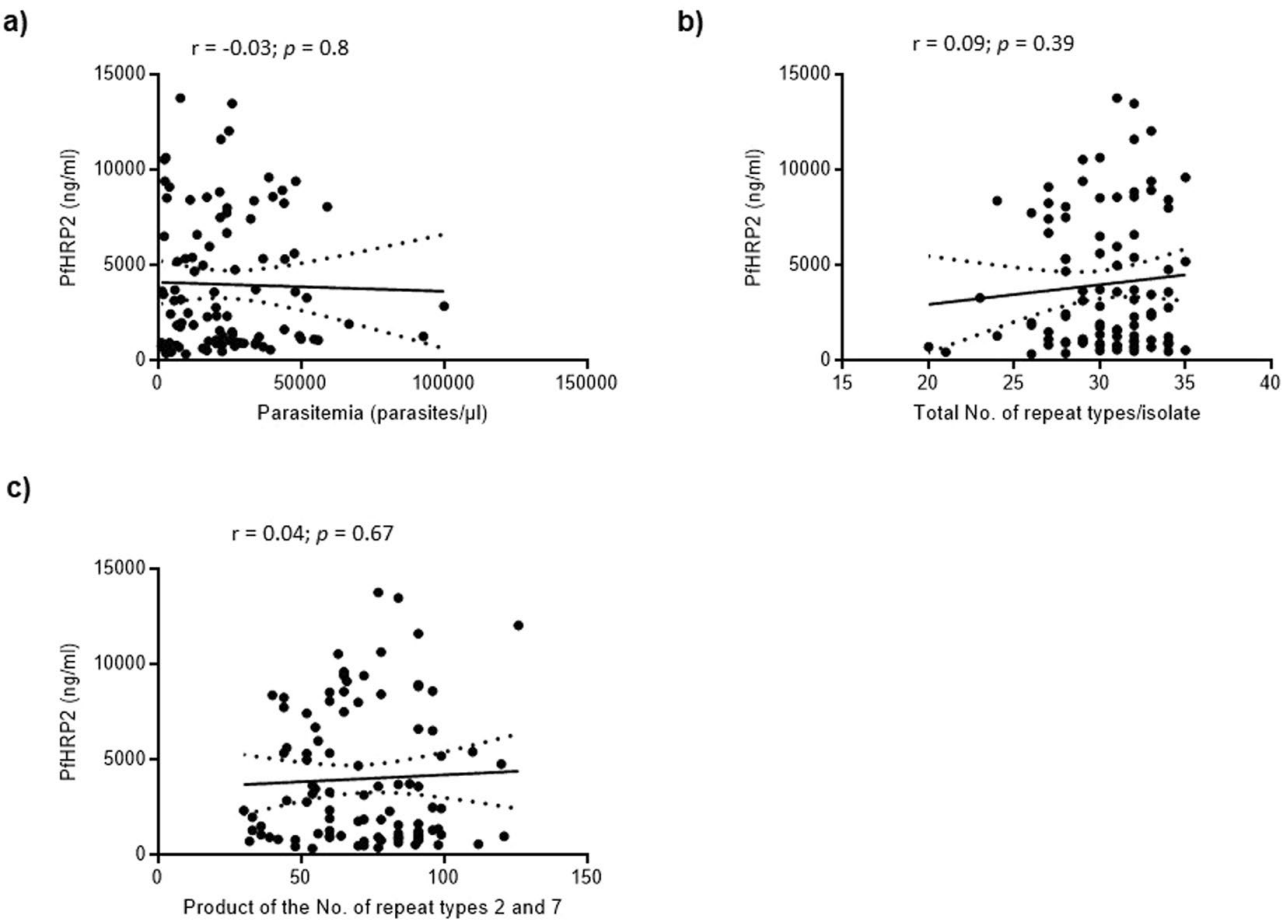
Results of Spearman’s correlation analysis between PfHRP2 levels and three *P. falciparum* parameters. (**a**) PfHRP2 vs. Parasitemia. (**b**) PfHRP2 vs. Total number of PfHRP2 repeat types per isolate. (**c**) PfHRP2 vs. Product of the number of repeat types 2 and 7. Statistical significance set at *p*-*value* < 0.05.

PfHRP2 levels were measured in samples from 96 uncomplicated malaria cases with a median parasite density of 21,400 parasites/µl (interquartile range, IQR: 7,781–34,180 parasites/µl) and a mean haemoglobin level of 10.48 g/dl (95% CI 10.13–10.82 g/dl). The concentration of PfHRP2 in whole blood among the participants was highly variable. It ranged from 339.3 ng/ml to 13,766 ng/ml with a median of 2,470 ng/ml (IQR: 980.8 ng/ml–6,670 ng/ml). PfHRP2 levels did not correlate with parasitemia, the number of individual PfHRP2 repeat types per isolate and the product of the number of repeat types 2 and 7 per isolate (Fig. 2).

## Discussion

PfHRP2 targeting RDTs are an important pillar of malaria control programmes and promote access to malaria diagnosis where microscopy is not available. Future use of these tests in malaria-endemic countries is threatened by the spread of isolates that do not express PfHRP2^18,24,25^. Since most of the commercially available RDTs target PfHRP2 expressed solely by *P. falciparum*, analysis of *pfhp2/3* genetic diversity is of public health importance.

We analysed the diversity of *pfhrp2* and *pfhrp3* among 400 isolates collected at five different time points (2007–2016) in Coastal and Western Kenya. Our study shows that *pfhrp2* and *pfhrp3* deletion did not occur among these isolates. This is consistent with two previous reports^16,17^. In 2017, however, Beshir *et al*. published the first report of *pfhrp2* deletion in Mbita, Kenya^15^. We analysed 58 samples collected from this area in 2007, seven years before the Beshir *et al*. sample collection in 2014. Absence of *pfhrp2/3* deletion in the present study strongly suggests that the reported *pfhrp2* and *pfhrp3* deletion may have occurred only recently in Mbita. It is important to note that the analysis of a small sample size in our study and the inclusion of symptomatic malaria cases may have influenced the results of this study. The latter is highly plausible considering that the Beshir *et al*. study analysed isolates obtained from asymptomatic malaria cases. Moreover, differences in the complexity of infection (COI) could have limited the detection of *pfhrp2/3* deletion as reported recently^26^.

At the amino acid sequence level, the structural organisation of repeat types was highly diverse. Ninety−four percent of the different PfHRP2 sequences identified in this study occurred only once. Similar findings have been reported in other malaria-endemic countries with Peruvian isolates being the least diverse^16,17^. Nevertheless, several characteristics were shared between isolates. Majority of the PfHRP2 sequences started with repeat type 1 and terminated with type 12, unlike in isolates from Senegal where type 12 was uncommon^27^. Similarly, 50% of the isolates had a previously described motif of repeat types 2, 3, 5, 7, 8, 2 and 7, which has been identified in 44% of *P. falciparum* isolates globally^16^. An additional 27% of our isolates had part of this motif (7, 8, 2 and 7), which is predominant in isolates from Madagascar^28^. The motif of types 2, 4, 5, 6, 7 and 8 found in Indian isolates was absent^13^.

In contrast to the diverse structural organisation described here, subtle differences were observed in the occurrence and number of PfHRP2 repeat types per isolate between and within Kenyan malaria-endemic sites. We found types 2 and 7 in all isolates, however, types 9 and 11 were completely absent in all isolates as reported elsewhere^13,16,27–32^. Type 4 was found in a few isolates (27%) and the rare types 13 and 14 were found in <8% of isolates^27,29–31,33^. Our data show for the first time the occurrence of type 19 (3 isolates) in Kenya. Other countries where type 19 was found are Uganda, Senegal, Mali and the Philippines^16,30,33^. The prevalence of the other repeat types identified were >80% consistent with earlier reports.

PfHRP3, on the other hand, showed lower variation than PfHRP2 in its structural organisation, occurrence and number of repeat types. This is reflected by the omnipresence of eight PfHRP3 repeat types identified previously^17^, the presence of a conserved repeat type organisation and a lower proportion (65%) of different PfHRP3 repeat type profiles. The identification of type 2 in the PfHRP3 of one isolate from Nyando confirmed our previous observation of the occurrence of this repeat in one isolate from Busia, Western Kenya, during the evaluation of a malaria RDT^34^. This strengthens our earlier hypothesis that type 2 presence in PfHRP3 may have occurred recently among Kenyan *P. falciparum* isolates. Beyond the Kenyan borders, the presence of type 2 in PfHRP3 has been reported from India at a prevalence of 2.9%^33^.

Another characteristic feature of histidine-rich proteins 2 and 3 from Kenyan isolates was the presence of repeat types that have not been described previously. Here, we identified 59 new repeat types arising from replacement of ≥1 amino acid of the previously described PfHRP2 and PfHRP3 repeat types. Majority of these new repeat types (39/59) were identified in PfHRP2, consistent with its higher diversity. A similar phenomenon was observed among isolates from the Chinese-Myanmar border where novel PfHRP2 repeat types originated from replacement of a single amino acid of eight amino acid repeats types, compared to the replacement ≥1 amino acid in 14 repeat types reported in this study^31^. Five additional repeats types (novel) have also been identified in Indian isolates^33^. Our study corroborates the existence of repeat types that are yet to be described and shows that they have a low prevalence. It also shows that there is a limited distribution of the new repeat types between different geographical regions. This is exemplified by the observation of the novel repeats types AHHAHHVAY (PfHRP2) and SHHDG (PfHRP3) among Kenyan isolates that were previously found in isolates from the China-Myanmar border and India, respectively^31,33^. Nevertheless, the new PfHRP2 repeat type AHHAAH (6%) and the new PfHRP3 repeat type SHHDG (10%) were most frequent in this study.

Currently it is of major importance in how far PfHRP2 diversity may affect malaria diagnoses based on the detection of PfHRP2. Baker *et al*. demonstrated, using a binary logistic regression model (Baker model), that the observed inter-study sensitivity variation of PfHRP2-based RDTs is linked to the product of the number of type 2 and type 7 (type 2 × type 7), especially with parasite densities of ≤250 parasites/µl^17^. In 2010, Baker *et al*. showed that RDT sensitivity does not correlate with type 2 × type 7 of isolates from different geographical areas^16^. Studies by Kumar *et al*. (2012) and Wurtz *et al*. (2013) have, however, associated type 2 × type 7 below 43 (group C) with RDT false negativity and reduced limited of detection, respectively^13,14^. This study was not able investigate this relationship in Kenyan isolates owing to the lack of PfHRP2-based RDT testing of the samples analysed here. Nevertheless, we utilized PfHRP2 classification of isolates based on type 2 × type 7 to determine the distribution of Kenyan isolates on basis of PfHRP2 diversity^17^. Our data reveals that most of Kenyan isolates (76%) are in group B (type 2 × type 7; ranges from 50 to 100). This finding is congruent with studies from Madagascar (69%) and Senegal (71%)^27,28^ and is comparable to our recent observation of 71% group B isolates in a small size of 38 samples^34^.

PfHRP2-specific monoclonal antibodies have been reported to also detect PfHRP3, a PfHRP2 homologue^17,35^. This cross-reactivity originates from the presence of repeat types 1, 2, 4 and 7, which are also found in PfHRP2. Consistent with this observation, we identified repeat types 1, 4 and 7 in PfPHP2 and PfHRP3 of Kenyan isolates apart from one isolate that exhibited these repeat types in addition to type 2. This implies that PfHRP3 may be useful in modulating the impact of PfHRP2 antigenic polymorphism in the context of malaria diagnosis using PfHRP2-based RDTs^15,36,37^. Whether cross-reactivity offers a diagnostic advantage remains to be validated due to the current lack of PfHRP3-specific monoclonal antibodies.

Along with genetic deletion and diversity of histidine-rich proteins 2 and 3, suboptimal antigen levels can contribute to false negative results of RDTs^38^. We observed a significant variation of PfHRP2 concentrations among uncomplicated malaria cases from Busia County, Kenya. Furthermore, the level of PfHRP2 did not correlate with parasitemia or the number of PfHRP2 repeat types per isolate. While we could not assess RDT sensitivity directly, our findings suggest that repeat type is not likely to be associated with RDT sensitivity, as previously observed by Baker *et al*.^16^. A previous study demonstrated, *in vitro*, that PfHRP2 expression varies between strains, the erythrocytic stages of *P. falciparum* and the mature schizonts account for most of the PfHRP2 released^39^. This presents a methodological challenge when investigating factors influencing PfHRP2 levels in the host. Additional confounding factors include PfHRP2 antibody cross-reaction with PfHRP3, PfHRP2 expression by gametocytes, slow antigen clearance and residual PfHRP2 from previous infections^6,40–42^. Nevertheless, the potential impact of PfHRP2 levels on sensitivity needs also to be considered during the evaluation of RDTs.

Taken together, this study shows extensive diversity of *Plasmodium falciparum* histidine-rich proteins 2 and 3 in Kenyan isolates. It also highlights the existence of additional amino acid repeat types which extends PfHRP2 and PfHPR3 antigenic variability. Information from this study will be useful to understand the performance of PfHRP2-detecting RDTs in this setting.

## Methods

### Study sites and sample selection

**Figure 3:**
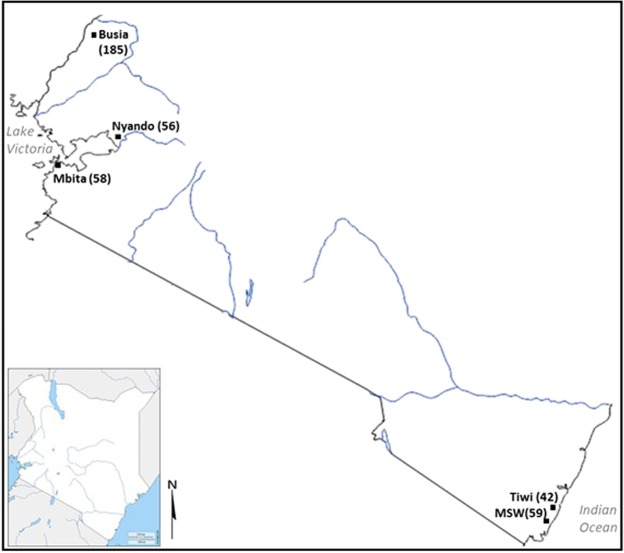
Map of Kenya showing the geographical location of the study sites. MSW is an abbreviation for Msambweni. The number of *P. falciparum* isolates recruited per study site shown in parentheses.

Four hundred *P. falciparum* isolates collected from symptomatic malaria cases in the frame of antimalarial drug efficacy trials conducted between 2007 and 2016, in malaria-endemic sites situated in Western (Mbita 2007, Nyando 2015 and Busia 2016) and Coastal (Tiwi 2008 and Msambweni 2013) Kenya were analysed in this study (Fig. 3). An inclusion criterion of >2,000 to 200,000 parasites/µl by microscopy was used in these studies. Samples were obtained before antimalarial drug administration and stored as dried blood spots (DBS) on filter papers, except isolates collected in Busia (whole blood in EDTA). Samples were included in the study after *P. falciparum* infection was confirmed using a *P. falciparum*-specific 18 S ribosomal RNA (rRNA) nested PCR.

### Ethical Statement

The study was approved by the Scientific & Ethics Review Unit (SERU) of Kenya Medical Research Institute (KEMRI) Nairobi (KEMRI/SERU/0152/3250) and written informed consent was obtained from the parents/guardians of all children and from adult participants. All experiments were performed in accordance with relevant guidelines and regulations.

### Molecular analysis

Genomic DNA was extracted using QIAamp DNA mini kit (Qiagen, Hilden, Germany) following the manufacturer’s instructions. Individual PCRs targeting *P. falciparum* 18S rRNA, *pfhrp2* (exon 2) and *pfhrp3* (exon 2) were performed as described previously (Table S1)^17,43^. Briefly, 1 µl of DNA template was added into a master mix containing 1 × PCR buffer (Qiagen, Hilden, Germany), 200 µM of each dNTP, 1 unit of Qiagen Taq DNA polymerase (Qiagen, Hilden, Germany), and 100 nM of each primer in a total volume of 20 µl. Genomic DNA of three *P. falciparum* strains was used as controls, namely 3D7 (*pfhrp2*^+^ and *pfhrp3*^+^), Dd2 (*pfhrp2*^−^ and *pfhrp3*^+^) and Hb3 (*pfhrp2*^+^ and *pfhrp3*^−^).

*pfhrp2* and *pfhrp3* PCR products were purified using Sephadex^TM^ G-50 fine DNA grade (GE Healthcare, Buckinghamshire, UK) and sequenced using the BigDye Terminator v3.1 Cycle Sequencing Kit (Applied Biosystems, CA, USA) according to the manufacturer’s instructions. PCR products were sequenced in the forward and reverse direction. DNA sequence chromatograms were visually inspected to resolve discordant base-calling. BioEdit (http://www.mbio.ncsu.edu/BioEdit/bioedit.html) was used to assemble the nucleic acid sequences using *P. falciparum* 3D7 *pfhrp2* (PF3D7_0831800) and *pfhrp3* (PF3D7_1372200) DNA sequences as the references as well as deduce amino acid sequences of PfHRP2 and PfHRP3.

### PfHRP2 and PfHRP3 diversity

The diversity of PfHRP2 and PfHRP3 was described based on the frequency, occurrence and organisation of histidine-rich protein repeat types (numerically coded as repeat types 1 to 24) as described by Baker *et al*.^16,17^. The product of the number of PfHRP2 repeat types 2 and 7 (type 2 × type 7) was used to classify *P*. falciparum isolates into four groups, namely group A (very sensitive), group B (sensitive), group I (borderline) and group C (non-sensitive) when the number of type 2 × type 7 was above 100, ranged from 50 to 100, ranged from 44 to 49, and less than 43, respectively. This was conducted to determine the distribution of *P. falciparum* isolates based on PfHRP2 diversity.

### PfHRP2 quantification by enzyme-linked immunosorbent assay (ELISA)

The measurement of PfHRP2 levels in whole blood samples of uncomplicated malaria cases from Busia County was conducted using a commercial sandwich ELISA kit (Malaria Ag Celisa^TM^, Cellabs, Sydney, Australia) in accordance with the manufacturer’s instructions. The optical density (OD) was measured at 450 nm in a PHOmo reader (Autobio Diagnostics Co. Ltd, Zhengzhou, China). The OD cut-off level was set by calculating the mean OD + 3 SD of three negative controls included in each ELISA run. OD above and below the cut-off was regarded to be positive and negative, respectively. PfHRP2 concentration was calculated using a standard curve prepared using serial dilutions (1:1) of recombinant PfHRP2 in phosphate buffered saline (PBS).

### Data Analysis

Chi-square and Fisher’s exact tests were used to compare the occurrence of amino acid repeat types between and within malaria-endemic sites. Kruskal Wallis and Mann-Whitney U tests were applied for analysis of non-parametric data. Significance was observed at a *p*-*value* < 0.05.

## Supplementary information

Supplementary data on Primers and PCR conditions used

## Data Availability

Data analysed in this publication are available from the corresponding author on reasonable request. *Plasmodium falciparum* histidine-rich proteins 2 and 3 nucleic acid sequences are available in GenBank (*pfhrp2*: accession numbers MH230283 - MH230526; *pfhrp3*: accession numbers: MH230527 - MH230790).
