# Supplementary material for: Plasmodium falciparum histidine-rich protein (PfHRP2 and 3) diversity in Western and Coastal Kenya
Source: Sci Rep. 2019 Feb 8;9:1709. doi: 10.1038/s41598-018-38175-1 (PMC6368535; doi:10.1038/s41598-018-38175-1)
Supplement: Supplementary file 1 — Supplementary data on Primers and PCR conditions used [file 41598_2018_38175_MOESM1_ESM.docx]

***Plasmodium falciparum* histidine−rich protein (PfHRP2 and 3) diversity in Western and Coastal Kenya**

David Nderu^1,2^, Francis Kimani^3^, Kelvin Thiong’o^3^, Evaline Karanja^4^, Maureen Akinyi^3^ Edwin Too^3^, William Chege^3^, Eva Nambati^3^, Christian G. Meyer^1,5,6^, Thirumalaisamy P. Velavan^1,5,6,7^*

^1^ Institute of Tropical Medicine, University of Tübingen, Tübingen, Germany.

^2^ School of Health Sciences, Kirinyaga University, 10300, Kerugoya, Kenya.

^3^ Center for Biotechnology Research and Development, Kenya Medical Research Institute, Nairobi, Kenya.

^4^ Department of Biochemistry and Biotechnology, School of Biological and Life Sciences, Technical University of Kenya, Nairobi, Kenya.

^5^ Vietnamese-German Centre for Medical Research (VG-CARE), Hanoi, Vietnam.

^6^ Faculty of Medicine, Duy Tan University, Da Nang, Vietnam.

^7^ Fondation Congolaise pour la Recherche Médicale, Brazzaville, Republic of Congo.

*** Correspondence:**

PD Dr. Thirumalaisamy P. Velavan

Institute of Tropical Medicine

Wilhelmstrasse 27

72074 Tübingen, Germany

Phone: +49-7071-2985981

Fax: +49-7071-294684

E-mail: [velavan@medizin.uni-tuebingen.de](mailto:velavan@medizin.uni-tuebingen.de)

**Word count:** 3,571; Tables: 4; Figures: 3

**Supplementary tables**

**Table S1**: Primer sequences and PCR conditions for the detection of *pfhrp2* (*exon 2*), *pfhrp3* (exon 2) and *P. falciparum.*

| **Primer specificity** | **Primer name** | **Primer sequence (5'−3')** | **Cycling conditions** |
| --- | --- | --- | --- |
| *pfhrp2* | Pfhrp2-F1 | CAAAAGGACTTAATTTAAATAAGAG | 94°C for 5 min, followed by 40 cycles of [94°C for 50 s, 55°C for 50 s, and 70°C for 1 min] and final extension 72°C for 10 mins |
|  | Pfhrp2-R1 | AATAAATTTAATGGCGTAGGCA |  |
|  | Pfhrp2-F2 | ATTATTACACGAAACTCAAGCAC |  |
| *pfhrp3* | Pfhrp3-F1 | AATGCAAAAGGACTTAATTC |  |
|  | Pfhrp3-R1 | TGGTGTAAGTGATGCGTAGT |  |
|  | Pfhrp3-F2 | AAATAAGAGATTATTACACGAAAG |  |
| *Plasmodium* genus | rPLU5 | CCTGTTGTTGCCTTAAACTTC | 95°C for 5 min, followed by 30 cycles of [94°C for 1 min, 58°C for 2 min, and 72°C for 2 min] and final extension 72°C for 10 mins |
|  | rPLU6 | TTAAAATTGTTGCAGTTAAAACG |  |
| *P. falciparum* | rFAL1 | TTAAACTGGTTTGGGAAAACCAAATATATT |  |
|  | rFAL2 | ACACAATGAACTCAATCATGACTACCCGTC |  |

**NB**: F1 and R1 primers used to perform a single-step and primary PCR when DNA extracted from whole blood and dried blood spots were used as template, respectively. R1 and F2 primers used to perform a semi-nested PCR.
